# Genome-wide profiling of WRKY genes involved in flavonoid biosynthesis in *Erigeron breviscapus*

**DOI:** 10.3389/fpls.2024.1412574

**Published:** 2024-06-03

**Authors:** Wanling Song, Shuangyan Zhang, Qi Li, Guisheng Xiang, Yan Zhao, Fan Wei, Guanghui Zhang, Shengchao Yang, Bing Hao

**Affiliations:** ^1^ The Key Laboratory of Medicinal Plant Biology of Yunnan Province, National & Local Joint Engineering Research Center on Germplasms Innovation & Utilization of Chinese Medicinal Materials in Southwest China, Yunnan Agricultural University, Kunming, China; ^2^ Yunnan Characteristic Plant Extraction Laboratory, Kunming, Yunnan, China

**Keywords:** flavonoid, *Erigeron breviscapus*, WRKY, hormones, structure gene

## Abstract

The transcription factors of WRKY genes play essential roles in plant growth, stress responses, and metabolite biosynthesis. *Erigeron breviscapus*, a traditional Chinese herb, is abundant in flavonoids and has been used for centuries to treat cardiovascular and cerebrovascular diseases. However, the WRKY transcription factors that regulate flavonoid biosynthesis in *E. breviscapus* remain unknown. In this study, a total of 75 *EbWRKY* transcription factors were predicted through comprehensive genome-wide characterization of *E. breviscapus* and the chromosomal localization of each *EbWRKY* gene was investigated. RNA sequencing revealed transient responses of 74 predicted *EbWRKY* genes to exogenous abscisic acid (ABA), salicylic acid (SA), and gibberellin 3 (GA3) after 4 h of treatment. In contrast, the expression of key structural genes involved in flavonoid biosynthesis increased after 4 h in GA3 treatment. However, the content of flavonoid metabolites in leaves significantly increased at 12 h. The qRT-PCR results showed that the expression patterns of *EbWRKY11*, *EbWRKY30*, *EbWRKY31*, *EbWRKY36*, and *EbWRKY44* transcription factors exhibited a high degree of similarity to the 11 structural genes involved in flavonoid biosynthesis. Protein-DNA interactions were performed between the key genes involved in scutellarin biosynthesis and candidate *WRKYs*. The result showed that *F7GAT* interacts with EbWRKY11, EbWRKY36, and EbWRKY44, while *EbF6H* has a self-activation function. This study provides comprehensive information on the regulatory control network of flavonoid accumulation mechanisms, offering valuable insights for breeding *E. breviscapus* varieties with enhanced scutellarin content.

## 1 Introduction

*Erigeron brevisapus* is a traditional medicinal plant in the Asteraceae family and is mainly distributed in Southwest China. The sales revenue of traditional Chinese medicine preparations derived from *E. breviscapus* as a primary ingredient in China reached 3 billion RMB in 2020 (http://yn.chinadaily.com.cn/a/202007/27/WS5f1e9bb2a310a859d09da571.html). The main active flavonoid in *E. brevisapus*, scutellarin, is extracted from the leaves and has been extensively utilized in prescription injections for the treatment of cardiovascular diseases (Chen et al., 2021; Ju et al., 2021; Yang et al., 2022b; Zhang et al., 2022). We successfully elucidated the complete biosynthesis pathway of scutellarin and constructed the high-level production yeast factory (Liu et al., 2018; Wang et al., 2022). Recently, we reported the transcription factors that regulate scutellarin (R2R3-MYB) and anthocyanin (bHLH) biosynthesis in *E. brevisapus* as well (Gao et al., 2022; Zhao et al., 2022). However, the regulatory mechanism of the flavonoid pathway governed by the WRKY transcription factor family in *E. breviscapus* remains elusive.

WRKY transcription factor is the seventh largest TF family in higher plants and is named for its characteristic WRKY domain (Rushton et al., 2010). The typical structure of WRKY is the N-terminal, which contains conserved amino acid sequence WRKYGQK, whereas the C-terminal contains a zinc finger motif (C2H2 or C2HC) (Eulgem et al., 2000). According to the number of WRKY domains and the type of zinc finger motif, WRKY can be divided into three categories: Group I contains two WRKY domains, whereas Groups II and III have a single WRKY domain, WRKY domains of Group II and III family members are more similar in sequence to the C-terminal than to the N-terminal WRKY domain of Group I proteins (Eulgem et al., 2000; Dong et al., 2003). The members of Group II WRKY were further divided into five subgroups: IIa, including IIa, IIc, IId, and IIe, based on additional conserved structural motifs (Eulgem et al., 2000; Zhang and Wang, 2005). WRKY transcription factors play important roles in plant growth and development, defense regulation, stress, and synthesis of secondary metabolites (Eulgem et al., 1999; Johnson et al., 2002; Yu et al., 2012, Yu et al, 2013).

Since the first WRKY gene (*SPL1*) was cloned from sweet potatoes (Ishiguro and Nakamura, 1994), the identification and functional analysis of WRKY genes has developed rapidly in plants, especially in crops, fruits, and medicinal plants. Several WRKY transcription factors have been identified in *Arabidopsis thaliana*, *Glycine max*, *Vitis vinifera*, *Panax ginseng*, and *Salvia miltiorrhiza* (Wang et al., 2011; Guo et al., 2014; Li et al., 2015; Yang et al., 2017; Di et al., 2021). A total of 14549 WRKY genes were recorded in the Plant Transcription Factor Database (PlantTFDB) (Jin et al., 2017). Numerous studies have substantiated the close association between WRKY transcription factors and the biosynthesis of flavonoid metabolites (Amato et al., 2017; Duan et al., 2018; Wang et al., 2023). The overexpression of *AeWRKY32* (*Okra*) induced anthocyanin accumulation, with higher expression levels of *AtCHS1*, *AtCHI4*, *AtF3H1*, and *AtDFR2* in transgenic *Arabidopsis* (Zhu et al., 2023). *AtWRKY23* transcription factors regulate flavonol accumulation, auxin transport, root growth, and development (Grunewald et al., 2012). *VvWRKY70* and *NtWRKY11b* have been identified as regulators involved in flavonol biosynthesis, the content of flavonol significant decreased in *VvWRKY70*-overexpressing grape calli lines by inhibiting the promoter *VvCHS2*, *VvCHS3*, and *VvFLS4.* Conversely, overexpression of *NtWRKY11b* led to a substantial increase in flavonol content ranging from 37.8% to 80.7%. (Wang et al., 2021; Wei et al., 2023). *BcWRKY1* significantly increased the transcript of *CHS* to regulate flavonoid biosynthesis (Zeng et al., 2022). However, the current literature rarely reports on the regulatory mechanisms by which WRKY transcription factors regulate flavone and flavonol biosynthesis in medicinal plants.

Plant hormones have a prominent function in the modulation of the growth, development, reproduction, and secondary metabolism of plants, such as SA, ABA, GA3, and MeJA, shown to be involved in the regulation of flavonoid biosynthesis (Khan et al., 2015; Lucho-Constantino et al., 2017; Li and Ahammed, 2023). Exogenous ABA could promote the synthesis of ABA in *Artemisia argyi* leaves and up-regulated the content of chlorogenic acid, nevertheless significantly down-regulated other flavonoid metabolites after ABA treatment (Yang et al., 2022a). Exogenous GA3 evidently decreased the contents of naringin and naringenin in *P. chinense* Schneid seedlings (Yang et al., 2023). Recently, an increasing interest has focused on WRKY transcription factors response to plant hormones and involved in flavonoid metabolism (Schluttenhofer and Yuan, 2015; Vives-Peris et al., 2018; Xu et al., 2020; Yamamoto et al., 2020). *LrWRKY3* transcription factor response to MeJA may specifically interact with the *ANR* and *LAR* gene and might be involved in anthocyanins synthesis in *L. radiate*, regulated the content of pelargonidin-3-*O*-glucoside-5-*O*-arabinoside in *L. radiate* (Wang et al., 2023). *VqWRKY31* also activated SA defense signaling and changed the accumulation of stilbenes, flavonoids, and proanthocyanidins (Yin et al., 2022). Flavonoid compounds are involved in the defence of plants against biotic and abiotic stresses, WRKY transcription factors can respond to hormone signal transduction pathways, improve the accumulation of flavonoids, and play key roles in the regulation of various stressful stresses (drought, low temperature, wounds, disease-resistant, etc.) in plants (Pourcel et al., 2007; Agati et al., 2012; Han et al., 2018a, Han et al., 2018b). The main medicinal active ingredients of *E. breviscapu* are flavonoids, the study of the response mechanisms of WRKY transcription factors and flavonoids under hormonal stress, and can effectively analyze and identify WRKY transcription factors involved in the synthesis of scutellarin, and elucidate the molecular mechanisms by which WRKY transcription factors regulate the synthesis of scutellarin.

In this study, the conserved motifs, gene structure, chromosome location, phylogenetic trees, gene expression profile, and function of WRKY genes were identified based on the whole genome of *E. breviscapus*. Additionally, integrated metabolomic and transcriptomic analyses were performed to study the expression patterns of WRKY genes and flavonoid metabolites in response to exogenous hormone treatments. Our study revealed the expression of *EbWRKYs* and the accumulation of flavonoids differed under the treatment of three exogenous hormones in *E. breviscapus* leaves. We also identified three candidate WRKY genes potentially involved in the regulation of scutellarin biosynthesis. This study provided valuable guiding information for growth and development research and functional identification of WRKY transcription factors involved in scutellarin biosynthesis in *E. breviscapus*.

## 2 Methods

### 2.1 Plant treatment

The two-month-old *E. breviscapus* seedlings were germinated and cultivated in a growth chamber under controlled conditions at 22 °C with a photoperiod of 16 hours light and 8 hours dark. Furthermore, the leaves of *E. breviscapus* were treated with 200 mL of ABA, SA, and gibberellin 3 (GA3) solution at a concentration of 200 μmol/L. Leaf samples were collected at time points of 0 h, 4 h, 12 h, and 24 h, immediately frozen in liquid nitrogen, and stored at -80 °C. Each experimental sample has three biological repetitions.

### 2.2 Identification and physicochemical properties of WRKY proteins

The PfamScan v1.6 tool was employed to annotate the protein domains of the entire genome sequence of *E. breviscapus*, utilizing the Pfam 35.0 database. Sequences exhibiting E-values lower than Le-5 and encompassing the PF03106 domain were screened, while manually excluding any atypical characteristics observed in WRKY genes. The ProtParam tool (https://web.expasy.org/protparam/) was utilized for predicting various attributes of EbWRKY proteins, including molecular weights (MWs), isoelectric points (pIs), amino acid counts, open reading frame (ORF) lengths. Protein subcellular localization was predicted by PSORT (https://psort.hgc.jp/).

### 2.3 Protein domain and phylogenetic evolution analysis

Multiple sequence alignments were conducted using MAFFT v7.490 to elucidate the evolutionary relationship between *E. breviscapus* and *A. thaliana*. To investigate the interrelationship among *E. breviscapus* WRKY proteins, a phylogenetic tree encompassing both *E. breviscapus* and *A. thaliana* WRKY proteins was constructed through Phylipv3.698 software employing the neighbor-joining method with 1000 repetitions. Subsequently, EvolView (https://evolgenius.info/evolview/#/) was employed as an evolutionary tree visualization tool for further analysis. The WRKY protein sequences of *A. thaliana* were downloaded from the TAIR database (https://www.arabidopsis.org/).

### 2.4 Comprehensive analysis of WRKY genes

The intron-exon structures of the *EbWRKY* genes were determined using the gene structure display server provided by Peking University (http://gsds.cbi.pku.edu.cn/). The conserved motifs of WRKY proteins were predicted using MutipleEm for Motif Elicitation, a tool available at http://alternate.meme-suite.org/tools/meme. For motif prediction, we employed optimized parameters including any number of repetitions (20), minimum width (10), and maximum width (80). Gene structure and chromosome mapping analysis of WRKY family members were conducted using TBtools v1.098691, while collinearity between the WRKY gene family in *E. breviscapus* and *A. thaliana*, *Daucus carota*, *Helianthus annuus*, *Lycopersicon esculentum*, and *Solanum tuberosum* was analyzed with the one-step MCScanX tool. Collinearity within *E. breviscapus* was visualized using Advanced Circos software.

### 2.5 RNA-sequencing data analysis

The leaves of *E. breviscapus* were subjected to hormone treatment, followed by flash freezing in liquid nitrogen for RNA extraction and subsequent cDNA library construction. Transcriptome sequencing was performed using Illumina Hiseq 4000 platform. SkrTools (version 1.0) was employed to calculate the raw data generated from sequencing, which underwent filtration using Trimmomatic v0.39 and RiboDetector v0.2.4 (Bolger et al., 2014; Deng et al., 2022). Subsequently, rRNA sequences were eliminated from the raw data to obtain high-quality clean reads that were utilized for gene differential expression analysis.

### 2.6 Expression profiling analysis of *EbWRKY* genes in various tissues

The differential expression of *EbWRKYs* in roots, stems, leaves, and flowers was calculated using the Salmon software based on the previously assembled genomic data of *E. breviscapus*. Additionally, the expression levels in the transcriptome data treated with three exogenous hormones at different time points were analyzed. The TPM value (Transcripts Per Million) was used to calculate gene expression values. Clustering results and heat maps were generated using TBtools v1.098691 (Chen et al., 2020).

### 2.7 Metabolites analysis

The frozen and fresh leaves of *E. breviscapus* (100 mg) were ground in liquid nitrogen, and the homogenate was resuspended in pre-chilled 80% methanol and 0.1% formic acid by vortexing. The samples were incubated on ice for 5 min and then centrifuged at 15,000 g at 4°C for 20 min. The supernatant was diluted to a final methanol concentration of 53% for the LC-MS/MS analysis (Dunn et al., 2011). Samples were injected onto an Xselect HSS T3 column (2.1×150 mm, 2.5 μm) with a 20-min linear gradient at a 0.4 mL/min flow rate for the positive/negative polarity mode. The eluents used were eluent A (0.1% formic acid water) and eluent B (0.1% formic acid-acetonitrile). The solvent gradient was set as follows: 2% B, 2 min; 2-100% B, 15.0 min; 100% B, 17.0 min; 100-2% B, 17.1 min; 2% B, 20 min (Wang et al., 2014). The data files generated by HPLC-MS/MS were processed using the SCIEX OS Version.

The dried leaves of *E. breviscapus* powder sample (0.3 g) were dissolved in 50 mL of methanol, and the supernatant was extracted for 30 minutes by ultrasonic use for HPLC analysis. Samples were injected onto an Agilent EC-C18 column (4.6 x 100 mm, 2.7 μm), with a 50-min linear gradient at a 1 mL/min flow rate. The eluents used were eluent A (acetonitrile) and eluent B (0.1% phosphoric acid water). The solvent gradient was set as follows: 0-10 min, 12%- 15% A; 10- 32 min, 15% A; 32- 33 min, 15%- 20% A; 33- 50 min, 20%- 22% A. Scutellarin (SE), Chlorogenic acid (CGA), 3,5-dicaffeoylquinolinic acid (3,5-diCQA), and Erigoster B (EB) were quantified using the external standard method with standards purchased from Sigma-Aldrich (Shanghai, China). Variance significance analysis was conducted employing SPSS 20.0.

### 2.8 Co-expression network analysis

The expression of candidate *EbWRKYs* and key genes involved in flavonoid biosynthesis was extracted from the transcriptome data obtained from the roots, stems, leaves, and flowers of *E. breviscapus*. Initially, statistically significant correlations between differential metabolites were calculated using R (version 4.1.1). A significance level of *p*<0.05 was applied for statistical analysis. Subsequently, gene expression levels and relative metabolite contents were collected to identify correlation pairs with a Pearson product-moment correlation coefficient (PCC)≥0.6 and a *p*-value ≤ 0.05. The filtered genes were then utilized to construct a co-expression network which was visualized using Cytoscape version 3.3.0 software (https://www.cytoscape.org).

### 2.9 Protein-DNA interactions assays

The proteins-DNA interaction between EbWRKY11, EbWRKY36, EbWRKY44, *EbF6H*, and *F7GAT* was investigated using the bait construct pAbAi and prey construct PGADT7, which were generated through a BP reaction. The prey plasmid was transformed into the bait strain yeast Y1H and selected with supplemented medium containing SD/-Leu, SD/-Leu/Aba (Clontech). The binding domain was predicted using JASPAR (https://jaspar.elixir.no/).

### 2.10 Quantitative real-time PCR analysis

Leaves were collected, and total RNA was extracted from hormone-treated samples using a HiPure HP Plant RNA Mini Kit (R4165-02). Subsequently, cDNA synthesis was performed utilizing a PrimeScript RT Reagent Kit with gDNA Eraser (Takara, Japan). Gene-specific primers for qRT-PCR reactions were designed employing Primer3 web version 4.1.0 (https://primer3.ut.ee/) (**Supplementary Table S6**). A Quantstudio 5 Flex Real-Time PCR System (Thermo Fisher Scientific, USA) was employed to analyze three technical replicates. The expression levels of genes from different treatments were normalized to *EbACTIN2*. Finally, the relative expression levels were calculated using the 2^–ΔΔCt^ method and visualized using GraphPad Prism 8.0.2.

## 3 Results

### 3.1 Identification and physicochemical properties of WRKY genes in *E. breviscapus*

A total of 75 putative *EbWRKYs* were identified from *E. breviscapus* genomic data, which were designated as *EbWRKY1* to *EbWRKY75*. The number of amino acids ranged from 144 (*EbWRKY51*) to 756 (*EbWRKY19*), and the isoelectric points ranged from 4.97 (*EbWRKY29/47*) to 10.12 (*EbWRKY73*), including 45 acidic and 30 basic amino acids. In addition, the relative molecular weights ranged from 19.99 (*EbWRKY68*) to 80.89 kDa (*EbWRKY25*). Based on sequence analysis conducted using PSORT software, it was determined that the 75 *EbWRKY* proteins are localized within the nucleus, suggesting their potential regulatory roles as transcription factors in this cellular compartment. In addition, *EbWRKY19* is located on the cell membrane and may be involved in the expression and regulation of genes related to membrane transport (**Supplementary Table S1**).

### 3.2 Evolution and sequence analysis of WRKY transcription factors

**Figure 1:**
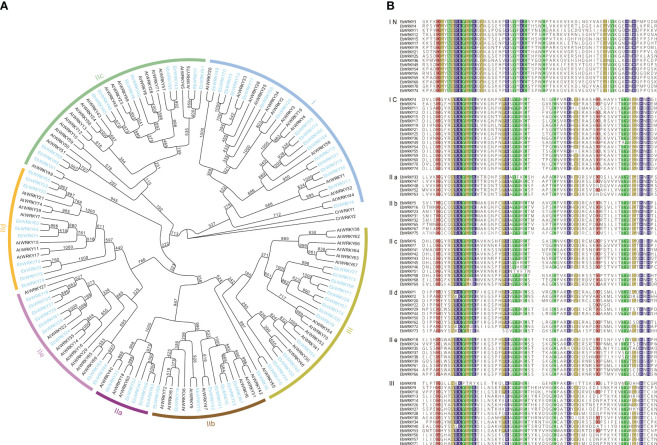
Phylogenetic and WRKY protein domain sequence analysis of *E. breviscapus*: **(A)** Phylogenetic analysis of *E. breviscapus* and *Arabidopsis* WRKY transcription factor; **(B)** WRKY protein domain sequence analysis in *E. breviscapus*. IN and IC represent the C-terminal and N-terminal WRKY domain of Group I, respectively.

To gain a comprehensive understanding of plant biodiversity mechanisms and the regulatory role of WRKY genes in the network, we conducted an evolutionary analysis of WRKY transcription factors. Subsequently, phylogenetic analyses were performed on 75 *E. breviscapus* and 72 Arabidopsis WRKY transcription factors (**Figure 1A**). A total of 147 *WRKYs* were divided into seven branches. *E. breviscapus* and *Arabidopsis* WRKY proteins with the same classifications were classified into I, II, and III. Group I has 17 *EbWRKYs*, Group II can be divided into five subtypes according to the different zinc-finger structural sites: IIa, IIb, IIc, IId, and IIe. There were five *EbWRKYs* in Group IIa, eight *EbWRKYs* in Group IIb, eleven *EbWRKYs* in Group IIc, ten *EbWRKYs* in Group IId, nine *EbWRKYs* in Group IIe, and fifteen *EbWRKYs* in Group III. The three major classes and five subclasses in the phylogenetic tree contained both the *WRKY* genes from *E. breviscapus* and *Arabidopsis*, indicating that the *WRKY* families of *Arabidopsis* and *E. breviscapus* are highly similar at the evolutionary level. Additionally, the WRKY transcription factors of *E.breviscapus* exhibit a high degree of similarity within their respective branches of the phylogeny, suggesting an increased homogeneity in the WRKY gene family during evolutionary processes.

In addition to WRKYGQK, the core motif of *E. breviscapus* WRKY heptapeptide contained five variants: WRKYGKK, WKKYGQK, WKKYGDK, WKKYGEK, and WSKYGQK (**Figure 1B**). Sequence comparison results showed that each WRKY protein, except *EbWRKY8*, contained a typical WRKY conserved domain at the N-terminal and a complete zinc finger structure at the C-terminal (CX4-5CX22-23HXH/C), which is an important feature for identifying WRKY transcription factors. All WRKY sequences of *E. breviscapus* showed a high similarity and conservation of the WRKY domain. Group I *EbWRKYs* contained the same heptapeptide core motif WRKYGQK at the N-and C-terminal and the zinc finger structure C2H2 behind the WRKY structure at the C-terminal. Group II and Group III had a WRKY domain at the N-terminal, but the zinc finger structure at the C-terminal differed (C2HX). *EbWRKY8* was domain sequence was lost, and the C-terminal retained a zinc-finger structure. However, the sequences of *EbWRKY8* are highly similar to the other *EbWRKYs*, and evolutionary analysis clustered them into Group III.

### 3.3 Gene structure and conserved motif analysis of WRKY proteins

**Figure 2:**
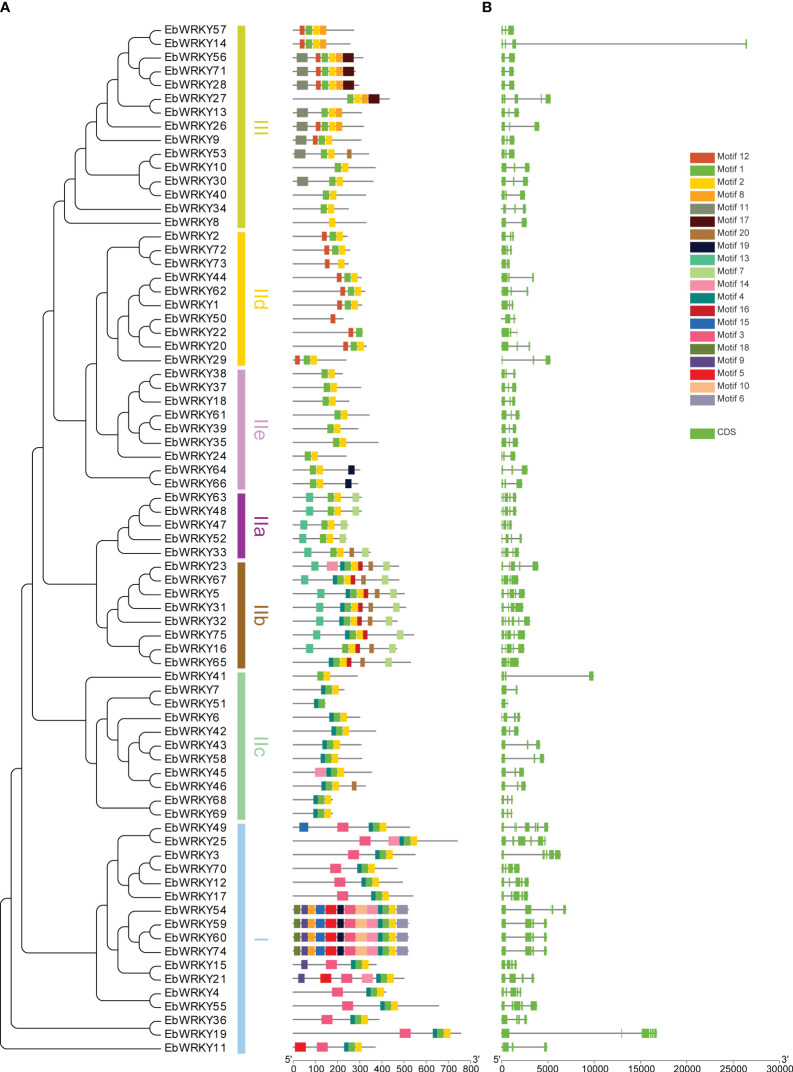
The evolutionary relationship, genetic structures, and motifs of WRKY genes in *E. breviscapus*: **(A)** Phylogenetic evolution and motif structure of *E. breviscapus* WRKY genes; **(B)** Gene structure of *E. breviscapus* WRKY genes (green box represents the exon).

75 WRKY protein motifs were analyzed using MEME and TBtools. The conserved domain of the WRKY motif was identified in motifs 1 and 3, while motif 2 exhibited a zinc-finger structure. Motifs 1 and 2 were found in almost all *EbWRKY* proteins, indicating their widespread presence. Notably, distinct *EbWRKYs* displayed diverse motif structures, with similar motifs observed within each branch clustering by the same type of *EbWRKY*. Motif 15 was exclusively present in the transcription factor genes of *EbWRKY49*, *EbWRKY54*, *EbWRKY59*, *EbWRKY60*, and *EbWRKY74* in Group I. Motif 16 was identified as a characteristic motif of Group IIb *EbWRKYs* while motif 4 was found to be a common feature of Groups I and II (b,c). Only eight members of Group III contained motif 11 whereas motif 12 was detected both in the sequences of Groups III and IId *EbWRKY* (**Figure 2A**; **Supplementary Table S2**). Phylogenetic trees of WRKY proteins were established, and the three groups were clustered according to their sequence similarity. WRKY sequences with similar structures in the evolutionary tree clustered into a single branch, indicating that these WRKY proteins may have similar functions. TBtools were used to analyze the number and distribution of exons of CDS sequences of the 75 WRKYs (**Supplementary Table S3**). The results showed a significant difference in the number of introns and exons in the WRKY gene family of *E. breviscapus*. The numbers of introns and exons were 1-6 and 2-7. (**Figure 2B**).

### 3.4 Chromosomal mapping and collinearity analysis of WRKY genes

**Figure 3:**
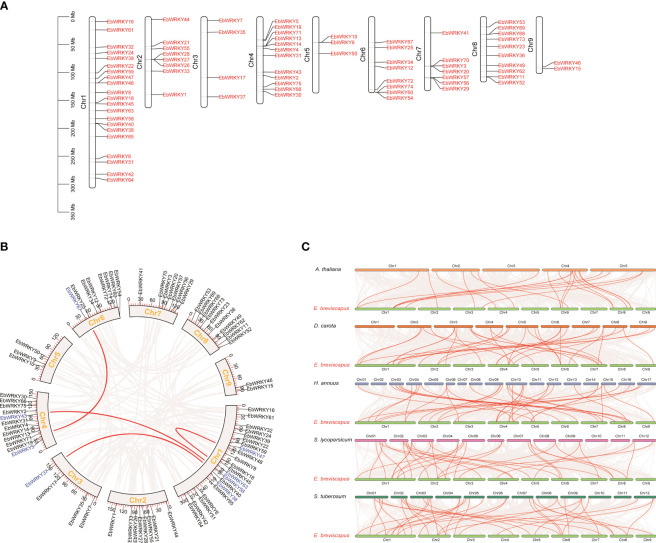
The Synteny analysis and chromosome location of WRKY genes in *E. breviscapus*: **(A)** Chromosomal location of WRKY genes; **(B)** The internal collinearity circle diagram of the *E. breviscapus* genome (the black line represents the position of genes on chromosomes; the arc represents the collinearity relationship between genes; the WRKY gene pair is highlighted with a red line; the gene name color represents different subgroups); **(C)** WRKY gene pairs are highlighted with red lines according to the analysis of collinearity between *E. breviscapus* and different plant genomes.

The distribution of the 75 *EbWRKY* genes across all nine *E. breviscapus* chromosomes exhibited irregular patterns (**Figure 3A**). A total of 21 *EbWRKY* genes were localized to chromosome 1, accounting for 28% of the *EbWRKY* gene family. Ten tandem duplications occurred on the six chromosomes. Eight WRKY members were collinear on chromosomes 1, 3, 4, and 6, respectively (**Figure 3B**). To further predict the potential evolutionary patterns of the *EbWRKY* gene family, we constructed comparative syntenic maps of *E. breviscapus* in associated with five representative species, including *A. thaliana*, *D. carota*, *H. annuus*, *L. esculentum*, and *S.tuberosum* (**Figure 3C**). The number of orthologous gene pairs between *E. breviscapus* and *A. thaliana*, *D. carota*, *H.annuus*, *L.esculentum*, and *S. tuberosum* were 21, 37, 33, 34, and 36, respectively (**Supplementary Table S4**). These results revealed that the identified orthologous events of *EbWRKY*-*HaWRKY* were considerably higher than those of other WRKY species based on their close evolutionary relationship. An extensive level of synteny conservation and an increased number of orthologous events in *EbWRKY*-*HaWRKY* indicated that *EbWRKY* genes in *E. breviscapus* shared a similar structure and function with *HaWRKY* genes.

### 3.5 Expression pattern of WRKY genes in the different tissues and hormone treatment

**Figure 4:**
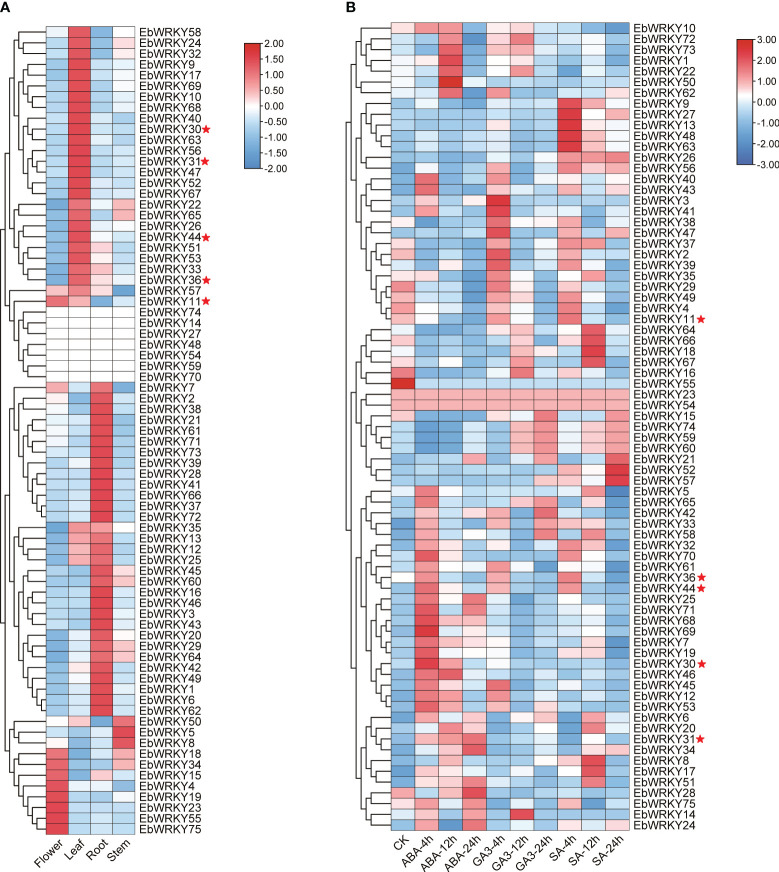
The expression profiles of WRKY genes in *E. breviscapus*: **(A)** Expression profiles of WRKY genes in different tissues of *E. breviscapus*; **(B)** Expression profiles of *E. breviscapus* WRKY under different hormone treatments at different times. The color scale on the right of each diagram represents TPM expression values: red indicates higher levels and blue indicates lower levels. CK represents the untreated sample. The red star represents five candidate *EbWRKYs* that may involved in the flavonoid metabolic pathway in *E. breviscapus*.

The TPM values of the roots, stems, leaves, and flowers were extracted from the genomic database to clarify the expression of *EbWRKY* family genes. Except for seven *EbWRKY* genes that exhibited no expression in any tissue, the remaining *EbWRKY* genes demonstrated specific expression patterns in leaves, roots, stems, and flowers, respectively. (**Supplementary Table S5**; **Figure 4A**). In our previous study, exogenous application of SA, GA3, and ABA onto the leaves of *E. breviscapus* was found to enhance their scutellarin (SE) content, with ABA treatment showing the most significant effect (**Supplementary Table S6**). Therefore, transcriptomic analysis was employed to investigate the underlying expression mechanism of *EbWRKYs* in response to hormone induction. The results showed that the expression levels of *EbWRKYs* significantly altered after three hormone treatments. In the ABA treatment, the expression levels of *EbWRKY69* and *EbWRKY30* were significantly up-regulated after 4 h. In contrast, in the SA treatment assays, the gene expression levels of *EbWRKY8*, *EbWRKY17, EbWRKY51, EbWRKY67, EbWRKY18, EbWRKY66*, and *EbWRKY64* were significantly up-regulated after 12 h of treatment, and *EbWRKY*52 and *EbWRKY57* were significantly up-regulated after 24 h. The expression levels of *EbWRKY3*, *EbWRKY41*, *EbWRKY47*, *EbWRKY2*, and *EbWRKY39* genes were significantly upregulated after 4 hours of GA treatment. However, a gradual decline in their expression levels was observed in the leaves over time. (**Figure 4B**).

### 3.6 Hormone-induced expression analysis of structural gene and flavonoid metabolites in the leaves *E. breviscapus*

**Figure 5:**
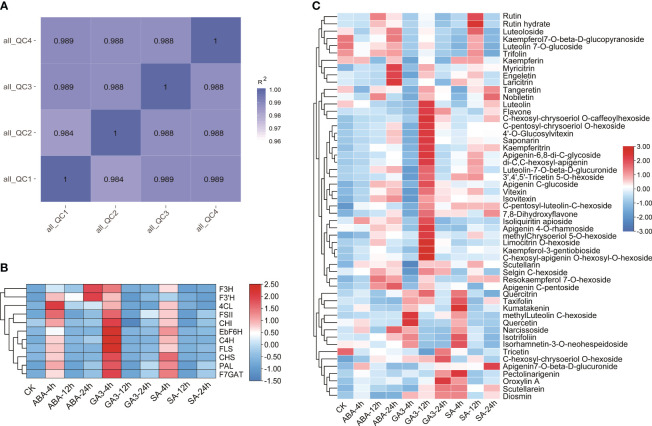
Quantitative and structural gene expression analysis of flavonoid pathway metabolites in the leaves of *E. breviscapus*: **(A)** Pearson correlation coefficient analysis of the QC of samples; **(B)** Structural gene expression analysis; **(C)** Contents of 54 flavone and flavonol metabolites in *E. breviscapus* under abscisic acid (ABA), salicylic acid (SA), and gibberellin 3 (GA3) hormone treatments at different times. CK represents the unprocessed sample.

Ultra-high-performance liquid chromatography (UPLC) and tandem mass spectrometry (MS/MS) were used to determine dynamic changes in flavonoid metabolites in nine *E. breviscapus* treated with the three hormones. Pearson correlation coefficients of the QC samples were calculated based on the relative quantitative values of the metabolites. The R^2^ values of all the samples were close to 1, indicating better stability of the entire detection process and higher data quality (**Supplementary Table S7**; **Figure 5A**). Scutellarin biosynthesis commences with phenylalanine, followed by the enzymatic catalyzation of phenylalanine ammonia-lyase (*PAL*), cinnamate 4-hydroxylase (*C4H*), coumaric acid coenzyme A ligase (*4CL*), chalcone synthase (*CHS*), chalcone isomerase (*CHI*), flavone synthase II (*FSII*), flavonoid 7-*O*-glucuronosyltransferase (*F7GAT*), and flavone-6 hydroxylase (*F6H*) to yield scutellarin. Furthermore, other flavonoids including kaempferol, quercetin hesperidin, and luteolin are biosynthesized via the flavanone 3-hydroxylase (*F3H*), flavonoid 3’-hydroxylase (*F3’H*), and flavonol synthase (*FLS*).

The expression patterns of 11 key enzyme genes involved in flavonoid biosynthesis were analyzed following treatments with ABA, SA, and GA3. The results showed that the expression of 11 genes treated with the three hormones was up-regulated at 4 h. The downstream genes regulating flavonol biosynthesis, *F3H* and *F3’H*, were significantly up-regulated in ABA treatment at 24 h but down-regulated in GA3 treatment. The gene expression of GA3 exhibited its peak at 4 hours, while the response to SA did not manifest prominently. The expression levels of *FLS*, *C4H*, and *F6H* were significantly up-regulated after 4 h of GA3 treatment (**Supplementary Table S8**; **Figure 5B**).

A total of 159 flavonoids were identified, with three biological replicates set for each sample (**Supplementary Table S7**). Flavones and flavonols accounted for the majority (57.8%), followed by flavanones (15.7%), isoflavones (10.6%), and anthocyanins (8.1%). Chalcones, dihydrochalcones (4.4%), and other flavonoids (3.1%) were found in lower abundance. The analysis revealed the identification of 92 flavone and flavonol metabolites, with scutellarin exhibiting the highest content, followed by Apigenin7-O-*β*-D-glucuronide (**Supplementary Table S7**; **Figure 5C**). After treatment with ABA, SA, and GA3, the metabolism patterns of 54 flavone and flavonol metabolites exhibited differential changes, and the responses of flavonol and flavone compounds significantly increased after 12 h of GA3 treatment, with 14 compounds significantly increased compared to other levels. In addition, the content of pectolinarigenin significantly increased after 24 hours of SA treatment, while the content of oroxylin A showed a significant increase after 24 hours of GA3 treatment compared to other levels.

### 3.7 Integrated analysis of WRKYs involved in flavonoid metabolism

**Figure 6:**
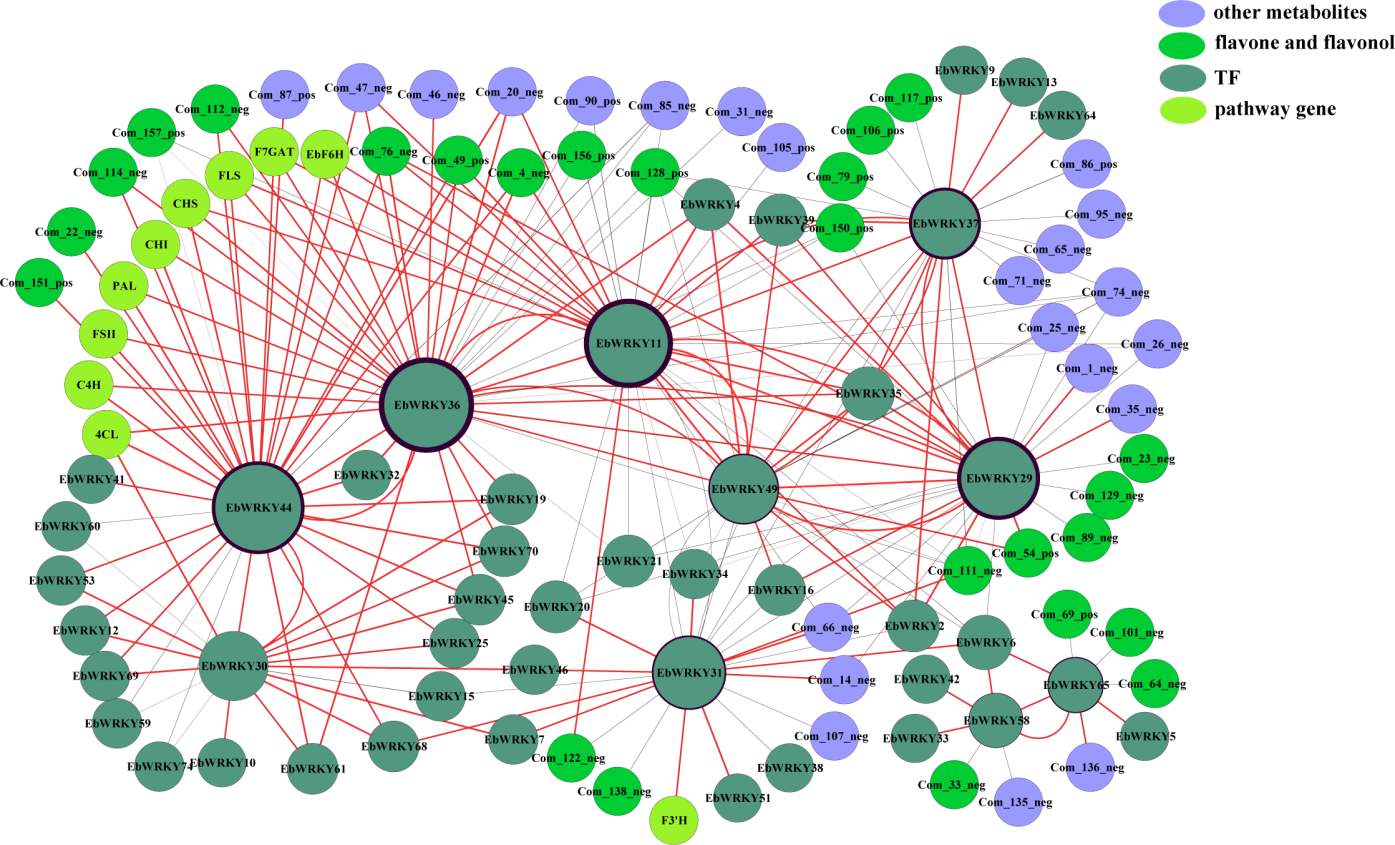
Co-expression analysis of structural genes involved in the flavonoid biosynthesis pathway and *EbWRKYs* in the leaves of *E. breviscapus*. Yellowish-green nodes represent genes; Blackish-green nodes represent WRKY TFs; Lavender nodes represent other flavonoid metabolites; Green nodes represent flavone and flavonol metabolites. The size of the circle is associated with the number of *EbWRKY* genes. Black circles outside the genes are associated with the number of metabolites.

Transcriptome and metabolome data were integrated and analyzed to construct a co-expression network of key genes involved in flavonoid metabolism pathways. A co-expression network was constructed by screening relevant pairs through Pearson analysis of gene expression levels and compound content (PCC ≥ 0.6, *p* ≤ 0.05). A total of 231 related pairs were identified and visualized using Cytoscape software (version 3.3.0). The network revealed a total of 102 interconnected nodes connected by 231 edges, encompassing 10 key enzyme genes and 45 *EbWRKYs*, alongside the presence of 47 flavonoid metabolites comprising 26 flavone and flavonol derivatives. In addition, a positive correlation was observed in 143 pairs, while 88 pairs exhibited a negative correlation (**Figure 6**). Within the flavonoid metabolic pathway, five potential *EbWRKYs* were identified as candidates with positive associations, namely *EbWRKY11*, *EbWRKY30*, *EbWRKY31*, *EbWRKY36*, and *EbWRKY44*. Notably, among these candidates, *EbWRKY11* demonstrated connections to four key genes (*EbF6H*, *F7GAT*, *FLS*, and *CHI*). The expression of *EbWRKY30* showed a positive correlation with *4CL*, *EbWRKY31*, and *F3’H*. Additionally, the presence of *EbWRKY36* and *EbWRKY44* was found to be associated with *PAL*, *EbF6H*, *C4H*, *4CL*, *F7GAT*, *CHI*, *CHS*, and *FLS.* Notably, *EbWRKY44* also exhibited a connection with *FSII.* (**Figure 6**).

Additionally, *EbWRKY11* exhibited association with a total of 17 flavonoid metabolites, encompassing nine flavone and flavonol metabolites. Among the 13 *EbWRKYs*, eight pairs displayed positive correlation while five pairs showed negative correlation. *EbWRKY31* was associated with six metabolites, including three flavones and flavonols. Seven pairs were positively correlated, and eight pairs were negatively correlated with 15 *EbWRKY*s. However, *EbWRKY30* was not directly connected with metabolites but was related to 17 *EbWRKY*s. The *EbWRKY36* gene was found to be associated with 18 flavonoid metabolites and 12 other *EbWRKY* genes, exhibiting positive correlations in 11 pairs and a negative correlation in one pair. The correlation analysis revealed that *EbWRKY44* was associated with 12 flavonoid metabolites and sixteen *EbWRKYs*, among which thirteen pairs exhibited positive correlations while three pairs showed negative correlations. Overall, the co-expression analysis of the selected *EbWRKY* genes revealed that these genes might play an essential role in flavonoid synthesis.

### 3.8 Quantitative real-time PCR profiling characterization of genes under exogenous hormone treatment

**Figure 7:**
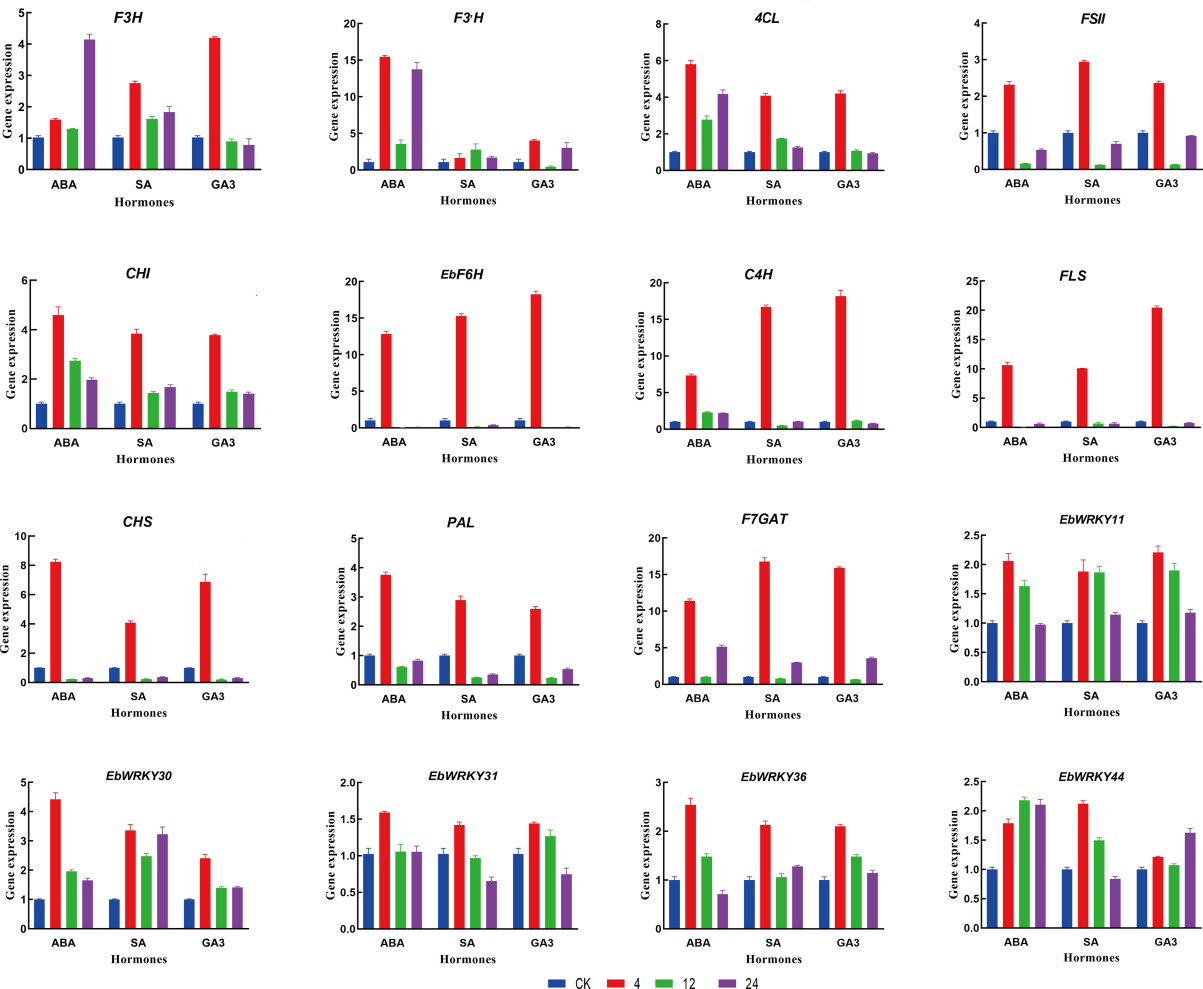
Relative expression of selected *Eb*genes in response to exogenous hormone treatment. Genes expression was analyzed by RT-qPCR. Blue was used as the untreated control (expression = 1); Red, green, and purple represent 4h, 12h, and 24h. Error bars represent standard errors. Data were calculated using the 2^–ΔΔCt^ method.

To investigate the expression pattern responses of genes under ABA, SA, and GA3 exogenous hormones in *E. breviscapus*, eleven structural genes of the flavonoid biosynthesis pathway and five WRKY genes (*EbWRKY11*, *EbWRKY30*, *EbWRKY31*, *EbWRKY36*, and *EbWRKY44*) were selected for qRT-PCR analysis after exogenous hormone treatment. Eleven genes involved in the flavonoid synthesis pathway exhibited significant up-regulation, implying their potential functional role in this biological process. (**Figure 7**; **Supplementary Table S9**). The relative expression of the selected key genes exhibited distinct temporal patterns in response to different treatments. Notably, *FLS* displayed the highest expression level, with a 20-fold increase observed after 4 hours of GA3 treatment and a 10-fold increase after 4 hours of ABA and SA treatment, followed by a subsequent decrease at 12 hours. *C4H, F6H*, and *F3H* showed similar expression patterns after 4 h of treatment, indicating that these genes are sensitive to GA3, SA, and ABA. *CHI* and *PAL* showed relatively high expression levels after 4 h of ABA treatment (> 3.5-fold). *CHS* exhibited the highest expression level, with an 8-fold expression at 4 h of ABA treatment and a 6.8-fold expression with GA3 treatment. *FSII* showed a 2.9-fold higher expression after 4 h of SA treatment.

The expression patterns of *EbWRKY11*, *EbWRKY30*, *EbWRKY31*, *EbWRKY36*, and *EbWRKY44* transcription factors related to the structural genes involved in flavonoid biosynthesis varied under different hormone treatments. *EbWRKY11* showed the highest expression level after 4 h of hormone treatment and gradually decreased after 12 h, indicating that *EbWRKY11* was sensitive to ABA, SA, and GA3. *EbWRKY30* was sensitive to ABA and had the highest expression level at 4 h with a 4.3-fold increase. In the SA treatment, an increase was followed by a decrease in the volatility of *EbWRKY30*. *EbWRKY31* showed a significant response to ABA treatment, and the expression level gradually decreased after SA and GA3 treatment at 4h. *EbWRKY36* exhibited a > 2-fold change in expression after 4 h of hormone treatments*. EbWRKY44* was more sensitive to SA than to ABA and GA3, with a 2.2-fold expression at 4 h. However, the gene expression level was highest at 12 h and 24 h of ABA treatment.

### 3.9 Protein-DNA interactions between EbWRKY11, EbWRKY36, EbWRKY44, and *F7GAT* and *EbF6H*

**Figure 8:**
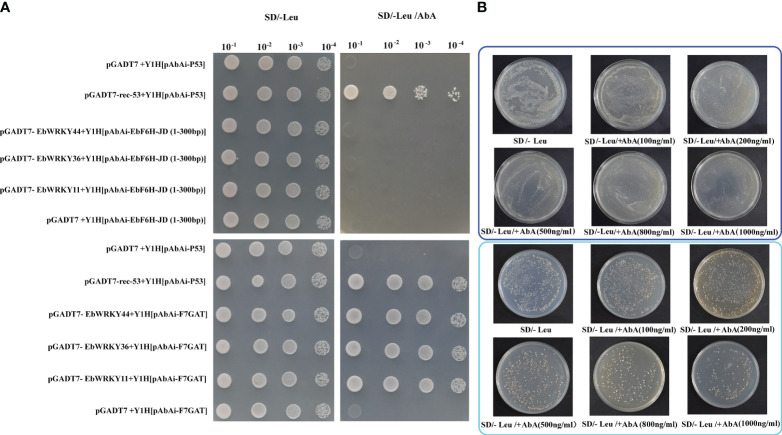
Protein-DNA interactions between EbWRKY11, EbWRKY36, EbWRKY44 with *EbF6H* and *F7GAT*. **(A)** Yeast One-Hybrid experiments. The pGADT7 was an AD empty vector; pGADT7-rec-53+Y1H[pAbAi-P53] was a positive control and pGADT7 +Y1H[pAbAi-P53] was a negative control. The EbF6H-JD (1-300bp) represents the promoter area of *EbF6H* 501-800bp. **(B)** *EbF6H* self-activation experiments. The upper blue box shows the full-length *EbF6H* promoter and the cyan box shows the area of 1501~2100bp promoter after the truncated *EbF6H*.

The yeast one-hybrid assay was conducted to validate the interaction between EbWRKY36, EbWRKY44, EbWRKY11 and two key structural genes, *F7GAT* and *EbF6H*, which encode key enzymes involved in the conversion of apigenin to scutellarin. The bait vector was constructed by utilizing the high GC% content of the *F7GAT* and *EbF6H* promoter domains. The results demonstrated that EbWRKY36, EbWRKY44, and EbWRKY11 exhibit binding affinity towards the promoter region *F7GAT* (1801-2500bp) (**Figure 8A**). Unfortunately, *EbF6H* was proven to have a self-activation function (**Figure 8B**). The two predicted regions with high scores containing WRKY-binding sites (ATAGTCAACT and TTCAAAGTCAAA) were truncated for verification. Notably, self-activation was observed in the 1501-2100bp region, while the 501-800bp region exhibited no self-activation but lacked interaction with the transcription factors EbWRKY36 and EbWRKY44, as well as EbWRKY11 (**Figures 8A, B**; **Supplementary Table S10**). These findings confirm that these three WRKY transcription factors of *E. breviscapus* play a role in the transcriptional regulation of key structural genes and regulated biosynthesis of scutellarin, a crucial active ingredient.

## 4 Discussion

The origins of WRKY transcription factors can be traced back to prokaryotes, with their presence limited to certain diplomonads, social amoebae, other amoebozoa species, and members of the fungal class incertae sedis (Rinerson et al., 2015; Chen et al., 2017). The 75 *EbWRKYs* were classified into two major branches: Group I was separated into a single branch, while the remaining *EbWRKYs* formed two complex branches consisting of individual sub-branches including Group IIc, Group IIa + IIb, Group IId + IIe, and Group III. Group II has the most numbers of *EbWRKYs*. Based on previous studies, WRKYs might evolve from the common ancestors, Group I WRKYs representing a more primitive form that subsequently evolved into Group II, encompassing subgroups IIa + IIb, IIc, and IId + IIe, Group III was closely related to Group IId and Group IIe (Zhang and Wang, 2005). In contrast, the present study revealed a closer genetic relationship between Group IIa + IIb and Group III compared to that between Group IId + IIe, suggesting a stronger evolutionary connection of *EbWRKYs* in Group IIa + IIb with those in Group III. These findings imply a shared ancestral origin among these genes.

The conserved domains of the *Eb*WRKY protein were further evaluated according to the motif characteristics of three categories and five subtypes. All WRKY protein sequences exhibited a completely conserved domain (WRKYGQK) and zinc finger structure associated with the WRKY motif except for *EbWRKY8*. The gene family phylogenetic analyses were consistent with the results of the motif structure and sequence alignment. The findings further substantiate that the evolutionary pattern of Group I WRKYs exhibits a higher degree of conservatism compared to other types, which was reported in previous study (Eulgem et al., 2000). Multiple sequence alignments indicated that, regardless of the common WRKYGQK heptapeptide sequence, there were other variations, mainly distributed in Group IIc (*EbWRKY68/69*) and Group IId (*EbWRKY2/72/73*). In addition, the WRKY domain variations of *EbWRKYs* occurred in Group III (*EbWRKY40*). The differences in the conserved domain of the WRKY protein may be caused by variations in the WRKYGQK heptapeptide sequence and zinc finger structure during evolution or the deletion mutation of amino acid residues (Zhang and Wang, 2005; Wang et al., 2011; Llorca et al., 2014). Mutations in the conserved domain reflect the diversity of the evolution of the plant WRKY gene family, similar minor variations have been observed in *Citrus* and *rice* (Xie et al., 2005; Ayadi et al., 2016). Variations in WRKYGQKs affect its affinity for the W-box and further influence its function (Eulgem et al., 2000; Maeo et al., 2001). Therefore, the interactions between *EbWRKY* proteins with these variations and downstream target genes, and their binding preferences with cis-acting W-box elements, should be further investigated.

The bioactive flavonoids, particularly scutellarin, are predominantly distributed in the leaves of *E. breviscapus*, which serves as the primary raw material for pharmaceutical extraction. In this study, 54 flavone and flavonol compounds showed spatiotemporal accumulation in the leaves of *E. breviscapus* after hormone treatments, notably, GA3 significantly improved the accumulation of flavones and flavonols in the leaves. Meanwhile, *EbWRKY30, EbWRKY31, EbWRKY36, and EbWRKY44* exhibited specific high expression levels in the leaves of *E. breviscapus*, while the expression level of *EbWRKY11* was lower than that in the roots but higher than in other tissues after hormone treatment. *EbWRKY44* and *EbWRKY36* exhibit significant increases in response to three hormones and treatment for 4 hours, while *EbWRKY30* and *EbWRKY31* specifically respond to ABA treatment only, displaying an inverse expression pattern of transcripts. These tissue-specific expression patterns suggest the potential involvement of these five *EbWRKY* transcription factors in flavonoid metabolism within leaves, while also indicating a possible role for *EbWRKY11* in root metabolism and development.

The WRKY transcription factor can bind to the promoter regions of functional genes or be induced by external stimuli, thereby regulating gene transcription levels involved in secondary metabolite accumulation (Liu et al., 2015; Bray, 1997; Shinozaki and Yamaguchi, 2000). Our previous work showed that *PAL*, *EbF6H*, *C4H*, *4CL*, *F7GAT*, *CHI*, *CHS*, *FLS*, *FSII*, *F3H*, and *F3’H* were key structural genes regulating flavonoid biosynthesis in the leaves of *E. breviscapus* (Gao et al., 2022; Zhao et al., 2022). *MdWRKY11* regulates anthocyanin synthesis through directly binding to the flavonoid 3-O-glycosyl transferase promoter in apple (Liu et al., 2019). *McWRKY71* controls *McANR* and proanthocyanidin synthesis in Malus crabapple (Zhang et al., 2022). Other reports demonstrated that F*aWRKY71* stimulates anthocyanin accumulation in strawberry (Fragaria×ananassa) by up-regulating *FaF3’H*, *FaLAR*, and *FaANR* (Yue et al., 2022). In this study, the structural genes of flavonoid biosynthesis of *E. breviscapus* significantly increased after 4 h of exogenous hormone treatment. The 11 structural gene expression patterns are basically consistent with the experimental results of qRT-PCR analysis in hormone treatment. *EbWRKY11*, *EbWRKY30*, *EbWRKY31*, *EbWRKY36*, and *EbWRKY44* are closely related to the genes of flavonoid biosynthesis. qRT-PCR analysis further verified the expression patterns of the structural genes involved in the flavonoid biosynthesis pathway are consistent with *EbWRKYs*. Therefore, these five *EbWRKY* transcription factors may participate in the transcription of key structural genes that regulate flavonoid metabolite accumulation.

The phylogenetic analysis revealed that *EbWRKY11*, *EbWRKY30*, *EbWRKY31*, *EbWRKY36*, and *EbWRKY44* were distributed across all three WRKY groups. The homologous genes of *EbWRKY44* in Group IId were *AtWRKY7/11/15/17* in *Arabidopsis*. In Group IIb, *AtWRKY*6, *AtWRKY31*, and *AtWRKY42* were co-orthologous to *EbWRKY31*. *EbWRKY30* in Group III was homologous to *AtWRKY30/46/41/53*. *EbWRKY36* and *EbWRKY11* were orthologs of *AtWRKY1* and *AtWRKY32*, which belonged to different nodes in the same branch of Group I. Previous studies have shown that WRKY transcription factors in *Arabidopsis* are widely involved in the regulation of biotic and abiotic stress, plant growth, and development. *AtWRKY15* regulates *Arabidopsis* growth and the salt stress response, whereas *AtWRKY7/11/17* are negative regulators of the PAMP immune system, which could enhance plant resistance to pathogens (Vanderauwera et al., 2012; Arraño-Salinas et al., 2018). In addition, *AtWRKY7* contains a CaM-binding domain, a new CaM-binding transcription factor that regulates plant growth and development and plays an important role in Ca^2+^signal transduction (Park et al., 2005). Moreover, *MxWRKY55* and *VvWRKY28* from Malus *xiaojinensis* and grape respectively, belong to Group II with WRKY TFs playing a role in plant resistance and contributing to higher salt tolerance (Han et al., 2020; Liu et al., 2022). *EbWRKY31* and *EbWRKY44* both belong to Group II and may be involved in salt stress response. Overexpression of *AtWRKY30* enhances abiotic stress tolerance in *A. thaliana* at the early growth stage (Scarpeci et al., 2013). *AtWRKY1* and *AtWRKY41* can resist *Pseudomonas syringae* (Mukhi et al., 2021). *AtWRKY42* regulates Pi homeostasis to adapt to environmental changes, and *AtWRKY6* participates in Pi transportation (Robatzek and Somssich, 2001; Su et al., 2015). *AtWRKY53* regulates stomatal movement and negatively regulates drought resistance, whereas *AtWRKY46* is involved in the sensitivity of *Arabidopsis* to drought and salt stress (Ma et al., 2017; Freeborough et al., 2021). Based on the evolutionary analysis results of *EbWRKYs* and *AtWRKYs*, it was speculated that *EbWRKYs* homologous to various branches of *Arabidopsis* might participate in plant growth, development, and responses to biological and abiotic stresses with other members in the branch.

Transcription factors can act alone or in conjunction with other proteins to form complex binding complexes at gene promoters, thereby exerting control over gene expression through physical interactions that span long distances and coordinate the transcriptional activation of specific genes (Banerji et al., 1981; Karin, 1990; Latchman, 1997; Yao et al., 2015);. In apple, *MdWRKY1* increases anthocyanin accumulation by activating *MdLNC499* and *MDERF109* expression (Ma et al., 2021). The interaction between *PyWRKY26* and *PybHLH3* targeting the promoter of *PyMYB114* may potentially modulate the accumulation of anthocyanins in red-skinned pears (Li et al., 2020). In this study, protein-DNA interactions between EbWRKY11, EbWRKY36, EbWRKY44, and *F7GAT* and *EbF6H*, which were typical glycosylase and hydroxylase involved in scutellarin biosynthesis in *E. breviscapus*. The results revealed that EbWRKY36, EbWRKY44, and EbWRKY11 demonstrate direct binding to the promoter of *F7GAT*, whereas these three *EbWRKYs* did not exhibit interaction with the promoter region of *EbF6H*(501-800bp). *EbF6H* cannot be directly regulated by *EbWRKY36*, *EbWRKY44*, and *EbWRKY11*, however, it has been demonstrated in grape that the indirect regulation of structure genes on flavonoid biosynthesis hydroxylation occurs through the regulation of other regulatory elements. *VvWRKY26* is preferentially recruited by a *VvMYB5a*-driven MBW complex to regulate flavonoid hydroxylation (Amato et al., 2019).

## 5 Conclusion

In this study, a total of 75 *EbWRKY* transcription factors were predicted from the genome of *E. breviscapus*. The amino acid number, molecular weight, predicted isoelectric point (PI) value, chromosome position, domain pattern, and conservative motif of *Eb*WRKYs were revealed by bioinformatics-based analyses. The specificity of *EbWRKYs* gene expression in different tissues and their expression pattern under hormone treatment were determined based on RNA sequencing. Combining metabolome and transcriptome results revealed the regulatory mechanism between the WRKY transcription factor and key genes involved in flavonoid biosynthesis. The expression patterns of *EbWRKY11*, *EbWRKY30*, *EbWRKY31*, *EbWRKY36*, and *EbWRKY44* transcription factors were similar to those of the 11 key structural genes involved in flavonoid biosynthesis. EbWRKY36, EbWRKY44, and EbWRKY11 can interact with the promoter of *F7GAT*, which was the key glycosyltransferase involved in scutellarin biosynthesis. We provided comprehensive information about the WRKY gene family of *E. breviscapus* and the mechanism of *Eb*WRKY genes involved in flavonoid metabolism regulation.

## Data availability statement

The raw data of E. breviscapus transcriptome in the current study are available in the National Center for Biotechnology Information (NCBI) database under project number PRJNA971382 (https://www.ncbi.nlm.nih.gov/bioproject/PRJNA971382). The genomic data of E. breviscapus is downloaded from the Medicinal Plants multi-Omics Database (http://medicinalplants.ynau.edu.cn/genome/detail/68).

## Ethics statement

Experimental research and field studies on plants comply with relevant institutional, national, and international guidelines and legislation, and all methods were performed according to the relevant guidelines and regulations. The cultivated *E. breviscapus* were collected with official permissions of Yunnan Hongling Biological Technology Co., LTD. Honghe, China.

## Author contributions

WS: Formal analysis, Writing – original draft. SZ: Data curation, Writing – original draft. QL: Software, Writing – review & editing. GX: Software, Writing – review & editing. YZ: Formal analysis, Project administration, Writing – review & editing. FW: Methodology, Writing – review & editing. GZ: Methodology, Writing – review & editing. SY: Funding acquisition, Investigation, Writing – review & editing. BH: Supervision, Writing – review & editing.
